# The Schott Methods of the Treatment of Chronic Diseases of the Heart

**Published:** 1895-12

**Authors:** 


*The Schott Methods of the Treatment of Chronic Diseases of the Heart.* By W. BEZLY THORNE, M.D. Pp. 80. London: J. & A. CHURCHILL. 1895.

In this book the treatment of heart diseases as conducted at Nauheim is carefully and lucidly described. A method of preparing artificial baths is also given, in addition to well-drawn illustrations of the various movements.

From actual observation at Nauheim we cannot doubt the value of the system in certain cases. The improvement indeed may be remarkable, but is possibly of not such wide application as some accounts might lead us to suppose. While no doubt valvular affections may materially improve, the cases most benefited appear to be cases of hypertrophy of the heart without valvular disease. At the end of the book Dr. Thorne gives ten illustrative cases, the majority of which appear to be of this nature.

We think that too great stress should not be laid upon the immediate reduction of cardiac dulness that follows immersion in the saline baths or after exercises. This we believe to indicate, not diminution in the size of the heart, but lung-distension. Dr. Thorne gives a diagram of the reduction in dulness in the case of a healthy heart after immersion in a saline bath. One generally considers that a healthy heart empties itself completely during systole; and if such be the case, a tepid bath must be unable to produce sufficiently great contraction to be detected as diminution of normal dulness. We examined a case before, during, and after a bath at Nauheim, and were surprised to find that marked diminution in the dulness took place. The impulse, however, remained beating in precisely the same spot—a fact that seemed to point conclusively to lung-expansion, not heart-contraction, as the reason for the diminution.

While, however, this detail is criticised, it must be again stated that the great benefit many patients receive cannot be doubted. The system is a most valuable addition to cardiac therapeutics, which the medical profession in England has to thank Dr. Thorne for bringing to their notice.

